# Gait stability in response to platform, belt, and sensory perturbations in young and older adults

**DOI:** 10.1007/s11517-018-1855-7

**Published:** 2018-06-27

**Authors:** S. Roeles, P. J. Rowe, S. M. Bruijn, C. R. Childs, G. D. Tarfali, F. Steenbrink, M. Pijnappels

**Affiliations:** 1 Department of Biomedical Engineering, University of Strathclyde, 106 Rottenrow East, G4 0NW, Glasgow, UK; 2 Department of Clinical Applications & Research, Motek BV, Hogehilweg 18-C, 1101 CD Amsterdam, The Netherlands; 3 Department of Human Movement Sciences, Faculty of Behavioural and Movement Sciences, Vrije Universiteit, Amsterdam Movement Sciences, De Boelelaan 1105, 1081 HV Amsterdam, The Netherlands; 4 Department of Orthopedics, First Affiliated Hospital of Fujian Medical University, Fuzhou City, 350005 Fujian China

**Keywords:** Accidental falls, Ageing, Margins of stability, Gait perturbations, Treadmill

## Abstract

Perturbation-based gait assessment has been used to quantify gait stability in older adults. However, knowledge on which perturbation type is most suitable to identify poor gait stability is lacking. We evaluated the effects of ipsi- and contra-lateral sway, belt acceleration and deceleration, and visual and auditory perturbations on medio-lateral (ML) and anterior-posterior (AP) margins of stability (MoS) in young and older adults. We aimed to evaluate (1) which perturbation type disturbed the gait pattern substantially, (2) how participants recovered, and (3) whether recovery responses could discriminate between young and older adults. Nine young (25.1 ± 3.4 years) and nine older (70.1 ± 7.6 years) adults walked on the CAREN Extended (Motek BV, The Netherlands). The perturbation effect was quantified by deviation in MoS over six post-perturbation steps compared to baseline walking. Contra-lateral sway and deceleration perturbations resulted in the largest ML (1.9–4 times larger than other types) and AP (1.6–5.6 times larger than other types) perturbation effects, respectively. After both perturbation types, participants increased MoS by taking wider, shorter, and faster steps. No differences between young and older adults were found. We suggest to evaluate the potential of using contra-lateral sway and deceleration perturbations for fall risk identification by including both healthy and frail older adults.

**Electronic supplementary material:**

The online version of this article (10.1007/s11517-018-1855-7) contains supplementary material, which is available to authorized users.

**Graphical abstract:**
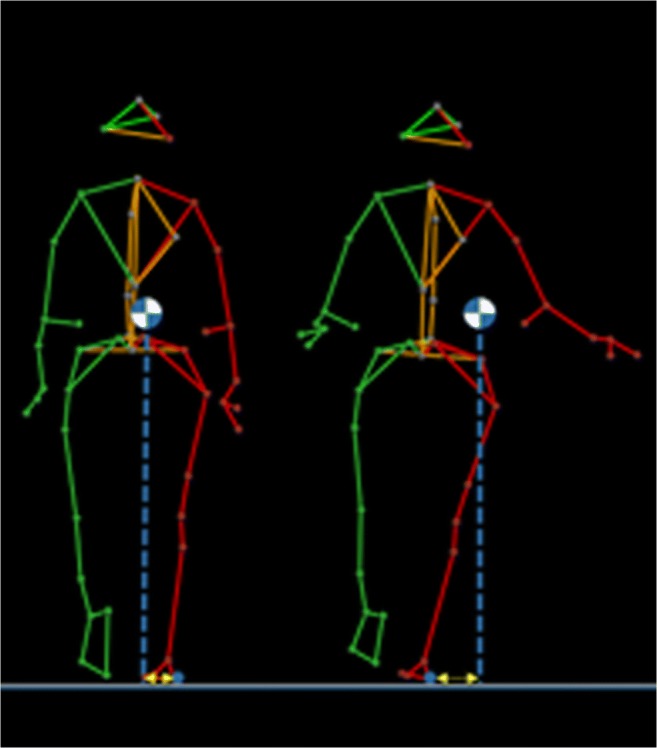
Margins of stability during steady state (left) and perturbed (right) gait to quantify reactive gait stability in response to various perturbation types in young and older adults.

## Introduction

Gait impairments are among the main risk factors for falls in older adults [1]. Since walking is one of the most common activities in our everyday life, it is not surprising that most falls occur while walking, due to trips or slips [2, 3]. Gait stability assessment to identify individuals at risk for falls is therefore of great importance [1]. Gait stability has been defined as “gait that does not lead to falls in spite of perturbations” and requires fast and accurate responses. However, the ability to respond adequately declines with age due to changes in the central nervous system and muscle properties [4]. Despite this knowledge, conventional balance and gait assessments solely evaluate self-initiated tasks (e.g., sit-to-stance transfers or turning). Such tasks allow for safe and controlled movement execution within one’s limits of stability. Recovering from gait perturbations, on the other hand, targets fundamentally different stability components. Therefore, it has emerged over the last few decades as a method to quantify gait stability in research, but not yet in clinical practice [5–9].

The majority of gait perturbation studies have included anterior-posterior (AP) perturbations using either moveable platforms [10, 11], obstacles [12–14] in an overground walkway, slippery surfaces [15], break-and-release systems [16, 17], or sudden treadmill belt accelerations and decelerations [18–21]. Additionally, medio-lateral (ML) perturbations have been applied by means of sideways platform movement [22–24] or waist-pulls [25–29]. Of less focus have been sensory perturbations, such as visual oscillations [30, 31] or low light conditions and distracting sounds [32–34]. Despite the growing body of work on the use of perturbations to evaluate one’s ability to resist or recover from a perturbation, it remains difficult to compare the wide range of applied methodologies and determine which perturbation type is appropriate for gait stability assessment.

The effect of perturbations on the gait pattern can be quantified by the ability to control the centre of mass (CoM) movement relative to the base of support (BoS) using measures like stabilizing and destabilizing forces, feasible-stability-region, and margins of stability (MoS) [35]. The latter is defined as the difference between the extrapolated centre of mass (XCoM; i.e. CoM position corrected for its velocity) relative to the border of the BoS. When the XCoM lies within the BoS one can be considered stable. In contrast, when the XCoM exceeds the border of the BoS, a corrective step needs to be taken to regain balance and avoid a fall; hence, one can be considered unstable [36]. In line with previous work, we quantified medio-lateral (ML) and anterior-posterior (AP) MoS using the lateral and backward border of the BoS, respectively [23]. As such, taking wider steps (i.e. stepping more lateral to the XCoM) results in larger ML MoS while faster and shorter steps (i.e. stepping more behind the XCoM) results in larger AP MoS [23]. Stepping responses to successfully recover from gait perturbations may provide valuable input for the development of tailored fall prevention training programs.

We developed a gait perturbation protocol, including six different perturbation types: two ML platform perturbations, two AP uni-lateral belt perturbations and two sensory (visual and auditory) perturbations, and tested it on healthy young and older adults. Our first aim was to evaluate which types of external perturbations affect the gait pattern the most in terms of ML and AP MoS, and as such, would be most suitable for perturbation-based gait stability assessment. Secondly, we identified how spatio-temporal adjustments were used to recover ML and AP gait stability. Finally, we evaluated whether these perturbation responses were sensitive to discriminate between young and older adults. Resulting knowledge can contribute to the design of an optimal experimental protocol that would have the best predictive value in identifying older adults at risk of falls (Table 1).

**Table 1:** List of abbreviations used in this study

1D	First (dominant) post-perturbation step
2ND	Second (non-dominant) post-perturbation step
3D	Third (dominant) post-perturbation step
4ND	Fourth (non-dominant) post-perturbation step
5D	Fifth (dominant) post-perturbation step
6ND	Sixth (non-dominant) post-perturbation step
6S	Total perturbation response
ANOVA	Analysis of variances
AP	Anterior-posterior
BoS	Base of support
CoM	Centre of mass
D	Dominant
ML	Medio-lateral
MoS	Margins of stability
ND	Non-dominant
NPD	Non-dominant pre-perturbation step
PD	Dominant pre-perturbation step
VT	Vertical
XCoM	Extrapolated centre of mass

## Methods

### Participants

Nine young adults (6 men and 3 women, age 25.1 ± 3.4 years, height 1.76 ± 0.09 m, weight 76.6 ± 15.1 kg) and nine healthy older adults (2 men and 7 women, age 70.1 ± 8.1 years, height 1.70 ± 0.11 m, weight 77.9 ± 10.5 kg) participated in this study. Inclusion criteria were normal lower limb function and being able to walk for 20 min. Exclusion criteria were neuromuscular deficits or weighing more than 135 kg. The Biomedical Engineering departmental ethics committee at the University of Strathclyde approved the protocol before measurements were performed. All participants gave informed consent prior to the measurement.

### Equipment

**Fig. 1:**
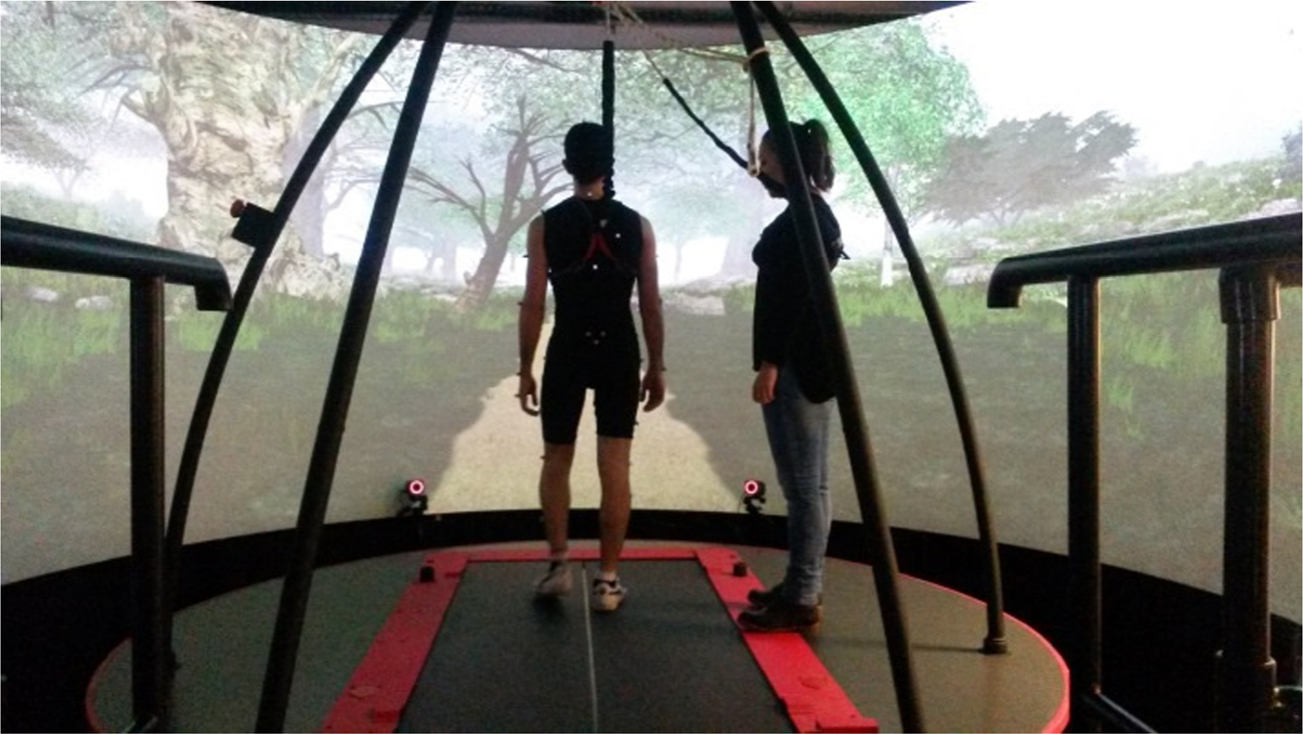
Photo of the experimental setup: the CAREN (Computer-Assisted Rehabilitation ENvironment, Motek, Amsterdam, The Netherlands)

Participants walked on the CAREN (Computer-Assisted Rehabilitation Environment) Extended (Motek, Amsterdam, The Netherlands) at the University of Strathclyde, which consists of a six degree-of-freedom motion base with an instrumented dual-belt treadmill mounted on top, 12 infra-red Vicon Bonita cameras (Vicon, Oxford, UK) operating at 100 Hz and a virtual reality environment projected on a semi-cylindrical screen and a surround sound system (Fig. 1). D-Flow software (version 3.20.0) was used to control all hardware components and to visualize the virtual environment [37]. The Human Body Model (Motek, Amsterdam, The Netherlands) containing 47 markers was used to calculate the body CoM [38]. Participants wore a safety harness to arrest potential falls.

### Protocol

First the participant’s dominant leg (preferred leg for kicking, climbing a stair and recovery from a push) was determined. Subsequently, comfortable walking speed was assessed by first gradually increasing treadmill speed until the participant had reached a comfortable speed. Speed was then further increased until participants reported to be uncomfortable. Thereafter, speed was gradually decreased until a comfortable speed was reached again. The treadmill speed was fixed to the average of the two reported comfortable speeds [39] after which a 3-min familiarization and a 2-min baseline trial were completed.

**Fig. 2:**
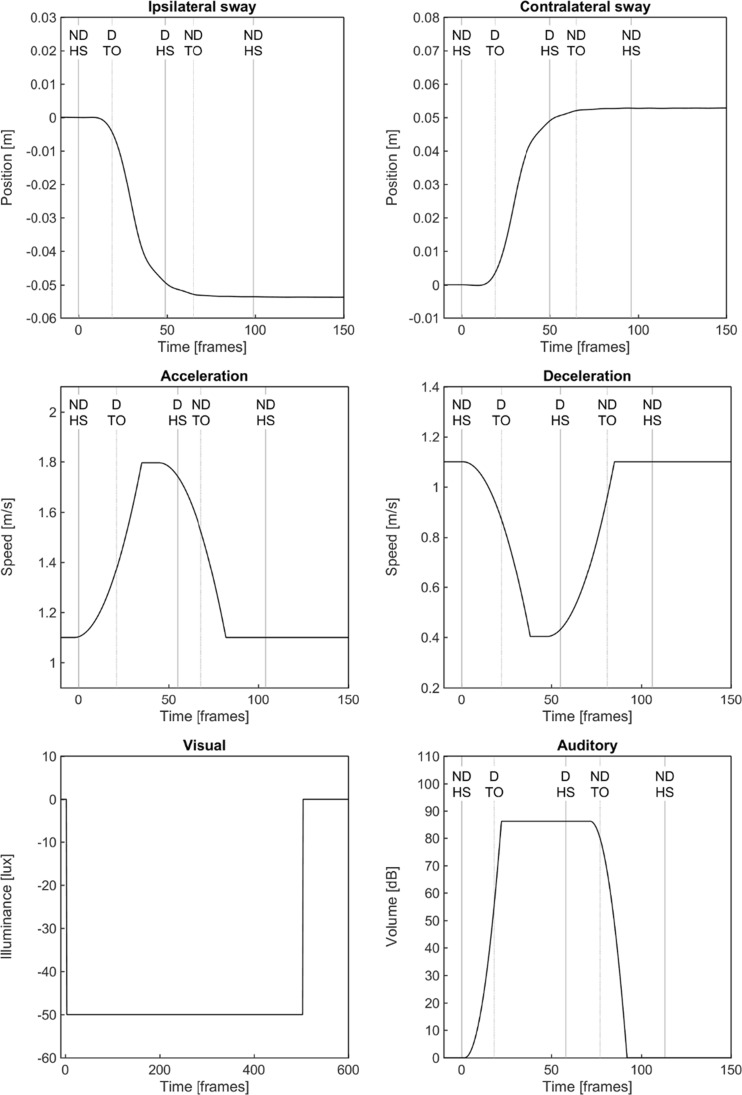
Perturbation profiles over time (recorded at 100 Hz) for ipsilateral sway, contra-lateral sway, acceleration, deceleration, visual and auditory perturbations. Dominant (D) and non-dominant (ND) heel strike (HS) and toe-off (TO) events are indicated by the vertical lines. All perturbations were triggered at the ND HS at Time = 0

The perturbation protocol contained six perturbation types all triggered at non-dominant heel strike [40]: (1) ipsilateral sway consisting of a 5-cm platform translation in approximately 0.7 s (maximum acceleration of 2.04 m/s^2^) to the non-dominant side, (2) contralateral sway which was identical to the ipsilateral sway perturbation but to the dominant side, (3) unilateral belt acceleration of the non-dominant side to 160% of the comfortable walking speed in approximately 0.4 s (maximum acceleration of 2.43 to 5.13 m/s^2^), (4) unilateral belt deceleration which was identical to the acceleration perturbation but with a minimum speed of 40% of the comfortable walking speed, (5) a visual perturbation by rapidly darkening the room for 5 s to < 1 lx, and (6) an auditory perturbation in the form of a 0.5-s lasting air horn at 82 dB (Fig. 2).

The protocol consisted of six trials, each consisting of one perturbation type which was repeated four times. The six trials were presented in random order. Ipsi- and contralateral sway trials always started with a ipsilateral and contralateral sway perturbation, respectively. The remaining perturbations were paired and presented in a pseudo-random order. This was necessary because the maximum platform excursion was 15 cm to each side.

### Data analyses

#### Outcome measures

All data were analysed using custom-written Matlab scripts (version 2015a; The Mathworks, Natick, MA, USA). First, marker data was filtered using a 6-Hz-second-order bidirectional Butterworth filter. Heel strike events were determined using the local maxima in the AP position of the heel marker relative to the pelvis [40].

Three spatio-temporal gait parameters were calculated: step time, step length, and step width. Step time, step length, and step width were defined as the elapsed time, AP distance and ML distance between two consecutive heel strikes, respectively.

**Fig. 3:**
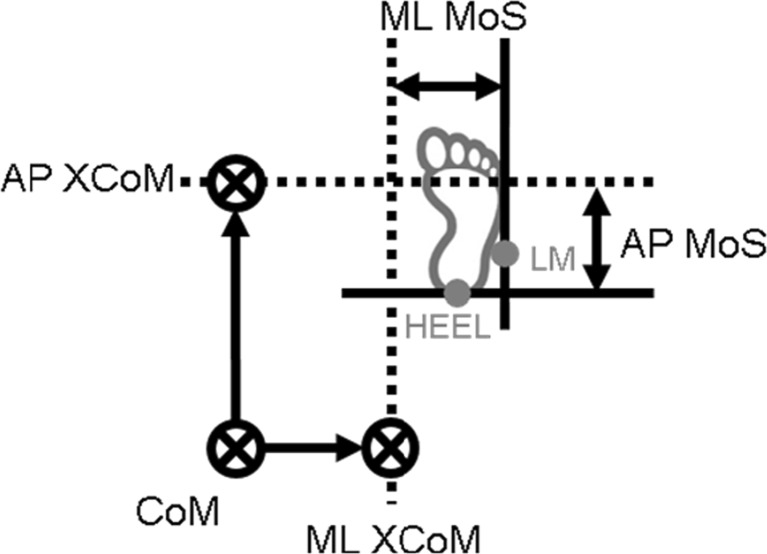
Schematic representation of margins of stability (MoS) for the right side in the medio-lateral (ML) and anterior-posterior (AP) direction

Gait stability was quantified by the MoS, as determined by the distance between the border of the BoS and the XCoM. The XCoM was estimated by the CoM position plus its velocity divided by $\sqrt{g/l}$ in which *g* is the acceleration of gravity and *l* the average greater trochanter markers’ height times 1.34 [36]. The ML lateral malleolus marker position of the leading foot quantified the ML border of the BoS whereas the AP heel marker position was used to define the AP border (Fig. 3). Thereby, negative ML and AP MoS values indicated *instability* in the lateral and backward direction, respectively.

Baseline values for spatio-temporal parameters and MoS were calculated and averaged over 100 consecutive dominant (BD) and 100 consecutive non-dominant (BND) steps. Additionally, local dynamic stability of ML, AP, and vertical (VT) trunk velocity over the same 100 strides was calculated as described in Bruijn et al. (2009) and used to evaluate unperturbed gait stability. Local dynamic stability reflects the ability to cope with small internal perturbations (e.g. variability in neuromuscular control) rather than external perturbations and has been used to detect age-related decline in steady state gait stability [41–43]. Lower local dynamic stability values imply more stable gait.

For the perturbation trials, spatio-temporal parameters and MoS were calculated for six steps pre- and six post-perturbation steps. To quantify which perturbation type affected the gait pattern the most, the difference of the six post-perturbation steps (1D, 2ND, 3D, 4ND, 5D, 6ND) with respect to BD and BND steps was calculated as:

$$ 6S=\sum \limits_{i=1}^2\sum \limits_{j=1}^3\sqrt{{\left(B(i)-P\left(i+\left(j-1\right)\ast 2\right)\right)}^2} $$

where *6S* is the deviation from baseline walking, *B* is baseline step for the *i*^th^ side (with *i* = 1 representing the dominant side and *i* = 2 representing the non-dominant side) and *P* is the post-perturbation step for *j*^th^ stride. We hereby captured the overall deviation from steady state walking while ignoring differences in recovery over subsequent steps. For example, a large initial deviation in step width but quick recovery to baseline values may result in similar 6S values as compared to a small initial deviation but slow return to baseline values.

Gait stability and stepping strategies in response to the perturbations were analysed by comparing average dominant pre-perturbation steps (PD) to dominant post-perturbation steps (i.e. 1D, 3D, 5D) and average non-dominant pre-perturbation steps (NPD) to non-dominant post-perturbation steps (i.e. 2ND, 4ND, 6ND). All perturbation measures were averaged over the last three perturbations of each perturbation trial.

### Statistical analyses

All statistical analyses were performed using SPSS version 23 (SPSS Inc., Chicago, IL, US). Gait parameters were tested for normality using a Shapiro-Wilk test. Differences between young and older adults in steady-state gait stability (i.e. local dynamic stability) were analysed using independent *t* tests. To evaluate whether dominant and non-dominant gait parameters differed at baseline, a mixed-model analysis of variances (ANOVA) was used (within factors: two sides; between factor: group). To examine which types of gait perturbations affect the gait pattern the most in terms of ML and AP MoS, mixed-model ANOVAs (within: six perturbation types; between: group) were applied for the total perturbation response (i.e. 6S). Post hoc pairwise comparisons were then used to find the perturbation types that affected gait stability the most for ML and AP directions. Subsequently, recovery from the perturbation was evaluated by analysing individual post-perturbation steps (i.e. 1D-6ND). The data dictated that participants pro-actively adapted their gait in anticipation of subsequent perturbations. Therefore, we first examined how participants adapted their gait by comparing baseline walking to pre-perturbation steps for all gait parameters using mixed-model ANOVAs (within: baseline and pre-perturbation step; between: group). Thereafter, to examine how participants recovered from the perturbations in terms of spatio-temporal parameters and ML and AP MoS, mixed-model ANOVAs (within: two pre-perturbation and six post-perturbation steps; between: group) for the individual steps were used. A Greenhouse-Geisser correction was used when the assumption of sphericity was violated. Post hoc paired-samples *t* tests with a Bonferroni correction for each perturbation type were used to investigate whether post-perturbation steps differed from pre-perturbation steps. The level of significance was set at 0.05.

## Results

All participants completed the protocol without falling. Mean comfortable walking speed (Y 1.26 ± 0.17 m/s, O 1.17 ± 0.23 m/s) did not significantly (*t* = 0.888, *p* = 0.388) differ between young and older adults.

### Baseline walking

Except for a larger dominant than non-dominant ML MoS (*t* = 5.702, *p* < 0.001) in both younger and older participants, the mixed-model ANOVA did not reveal any main or interaction effects when comparing dominant and non-dominant steps. Local dynamic stability was not significantly different between young and older adults in any direction (ML *p* = 0.835; AP *p* = 0.164; VT *p* = 0.516. See Table 2).

**Table 2:** Mean ± SD of baseline gait parameter for the dominant (BD) and non-dominant (BND) steps in young and older adults. Significant effects at *p* < 0.05 are printed in italics

Parameter		Young adults	Older adults	Main effect (sides)		Between subjects effect (Group)		Interaction effect (Steps × Group)	
		Mean ± SD	Mean ± SD	*F*	*p*	*F*	*p*	*F*	*p*
ML MoS [m]	D	0.065 ± 0.012	0.061 ± 0.013	*31.339*	*< 0.001*	1.218	0.286	0.388	0.542
	ND	*0.053 ± 0.006*	*0.047 ± 0.013*						
AP MoS [m]	D	0.169 ± 0.047	0.142 ± 0.052	2.888	0.109	1.729	0.207	1.009	0.330
	ND	0.175 ± 0.044	0.143 ± 0.045						
Step time [s]	D	0.544 ± 0.046	0.555 ± 0.033	0.015	0.905	0.285	0.600	0.113	0.741
	ND	0.545 ± 0.043	0.554 ± 0.032						
Step length [m]	D	0.680 ± 0.066	0.656 ± 0.125	2.488	0.134	0.430	0.521	2.113	0.165
	ND	0.692 ± 0.066	0.656 ± 0.113						
Step width [m]	D	0.126 ± 0.032	0.113 ± 0.056	1.539	0.233	0.356	0.559	0.293	0.596
	ND	0.126 ± 0.032	0.113 ± 0.056						
LDS_ML_	–	1.829 ± 0.294	1.798 ± 0.334	–	–	–	–	–	–
LDS_AP_	–	1.585 ± 0.263	1.404 ± 0.262	–	–	–	–	–	–
LDS_VT_	–	1.826 ± 0.428	1.701 ± 0.366	–	–	–	–	–	–

### Which perturbation type affected the gait pattern the most?

**Fig. 4:**
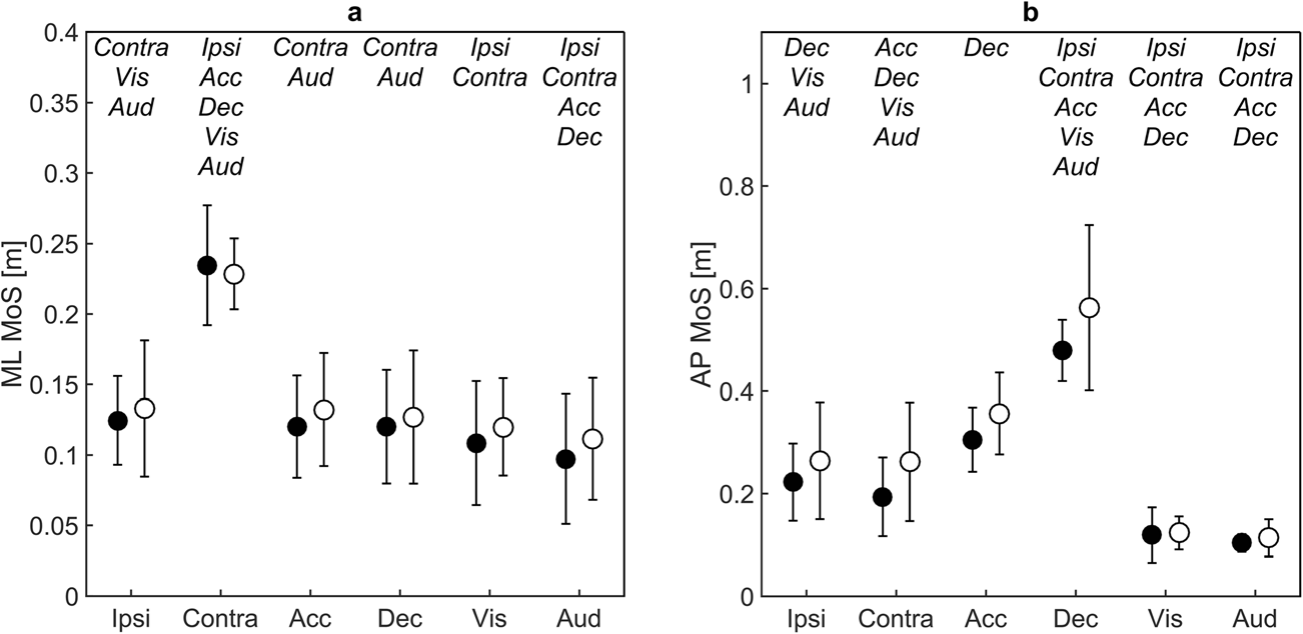
Mean and standard deviations of the overall perturbation effect 6S (see text for details) for medio-lateral (ML) and anterior-posterior (AP) margins of stability (MoS) after the ipsi-lateral sway (SwyI), contra-lateral sway (SwyC), acceleration (Acc), deceleration (Dec), visual (Viz) and auditory (Aud) perturbations. Black dots represent values for young adults whereas white dots represent older adults. Significantly different pairwise comparisons are indicated at the top per perturbation type in italic

The gait pattern was differently affected by the different perturbation types, without group or interaction effects (Main effects of perturbation for 6S ML MoS *F* = 76.023, *p* < 0.001, and for 6S AP MoS *F* = 85.281, *p* < 0.001). Post hoc pairwise comparisons revealed that 6S ML MoS in response to the contralateral sway perturbation was significantly larger compared to all other perturbation types meaning that ML MoS deviated most from baseline waking after the contralateral sway perturbation (mean difference 0.103–0.159 m; all at *p* < 0.001) (Fig. 4a). Similarly, 6S AP MoS was significantly larger for the deceleration perturbation compared to all other perturbation types (mean difference 0.287–0.430 m; all at *p* < 0.001) (Fig. 4b). Based on the significant effects of contralateral sway and deceleration on 6S ML and AP MoS respectively, these perturbation types were further investigated.

### How did participants adapt their gait in between perturbations?

Step width significantly increased prior to the contralateral sway perturbation compared to baseline walking for the dominant side (BD 0.120 ± 0.045, PD 0.127 ± 0.053 m, *F* = 4.830, *p* = 0.043) and a trend toward a significant increase was found for the non-dominant side (BD 0.120 ± 0.045, PD 0.127 ± 0.053 m, *F* = 4.150, *p* = 0.059).

AP MoS prior to the deceleration perturbation was significantly larger (i.e. more stable in the backward direction) compared to baseline walking for the non-dominant side (BD 0.166 ± 0.043, PD 0.181 ± 0.041 m, *F* = 11.709, *p* = 0.004) and near significant for the dominant side (BD 0.162 ± 0.047, PD 0.171 ± 0.044 m, *F* = 4.231, *p* = 0.059). Step width was significantly increased prior to the perturbation compared to baseline walking for both the dominant (BD 0.128 ± 0.038, PD 0.141 ± 0.044 m, *F* = 12.492, *p* = 0.003) and non-dominant (BD 0.129 ± 0.038, PD 0.143 ± 0.043 m, *F* = 10.119, *p* = 0.007) side.

### How were spatio-temporal adjustments used to recover ML and AP gait stability?

**Fig. 5:**
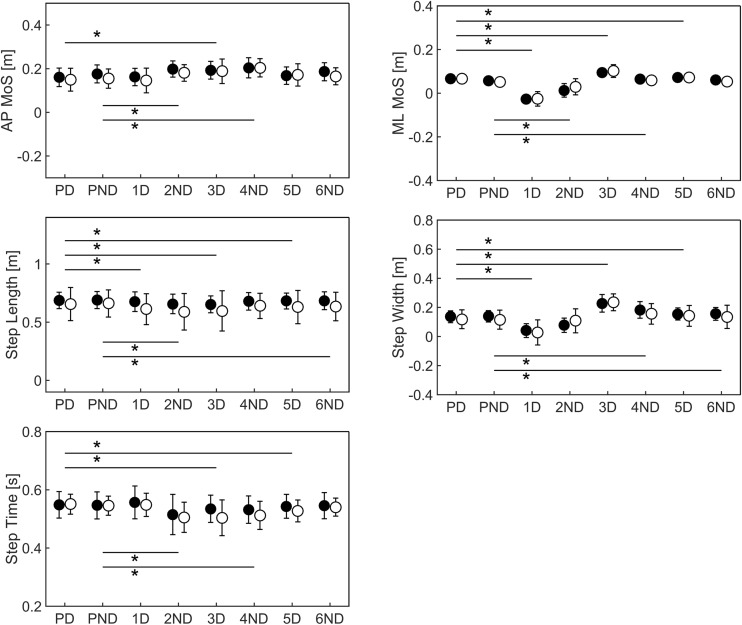
Mean and standard deviations of anterior-posterior (AP) and medio-lateral (ML) margins of stability (MoS), step length, width and time for steps for the contra-lateral sway (SwyC) perturbations. Black dots represent for values young adults whereas white dots represent older adults. Significant differences between pre-perturbation (PD and PND) and post-perturbation (1D to 6ND) steps are indicated with *

**Fig. 6:**
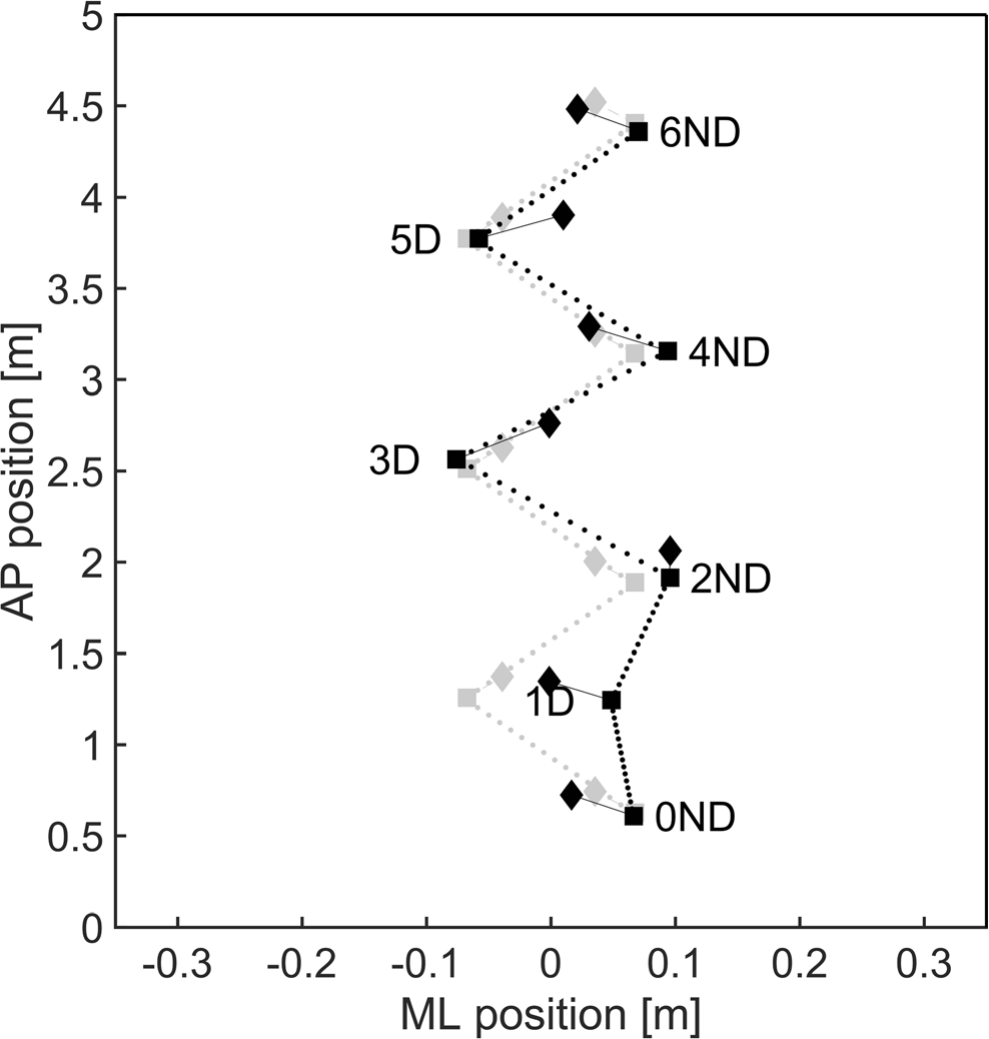
A schematic representation of a typical response (black) to a contra-lateral sway perturbation as compared to baseline walking (gray). Squares represent ML (step width) and AP (step length) foot placement whereas the line density is an indication of the time elapsed (step time) between consecutive steps. Margins of stability in the ML and AP direction are indicated by the diamonds. The perturbation was triggered at step 0ND

Mixed-model ANOVAs for the contralateral sway perturbation revealed significant main effects of Steps on all gait parameters while no significant Group or Group × Steps interaction effects were found. Post hoc analyses showed that step width and ML MoS were reduced at step 1D (Fig. 5 and Supplementary Material Table S1). Step width and ML MoS increased during step 2ND though ML MoS values remained smaller than at baseline. Thereafter, both parameters increased and remained larger compared to baseline walking. In other words, ML stability was initially compromised by the contralateral sway perturbation but was restored to values greater compared to baseline walking during the subsequent recovery steps. Step length (1D to 6ND) and step time (2ND to 5D) decreased, while AP MoS (2ND to 6D) increased meaning that participants became more stable in the backward direction. Figure 6 shows the relation between gait stability and spatio-temporal parameters for a typical response contralateral sway perturbation response.

**Fig. 7:**
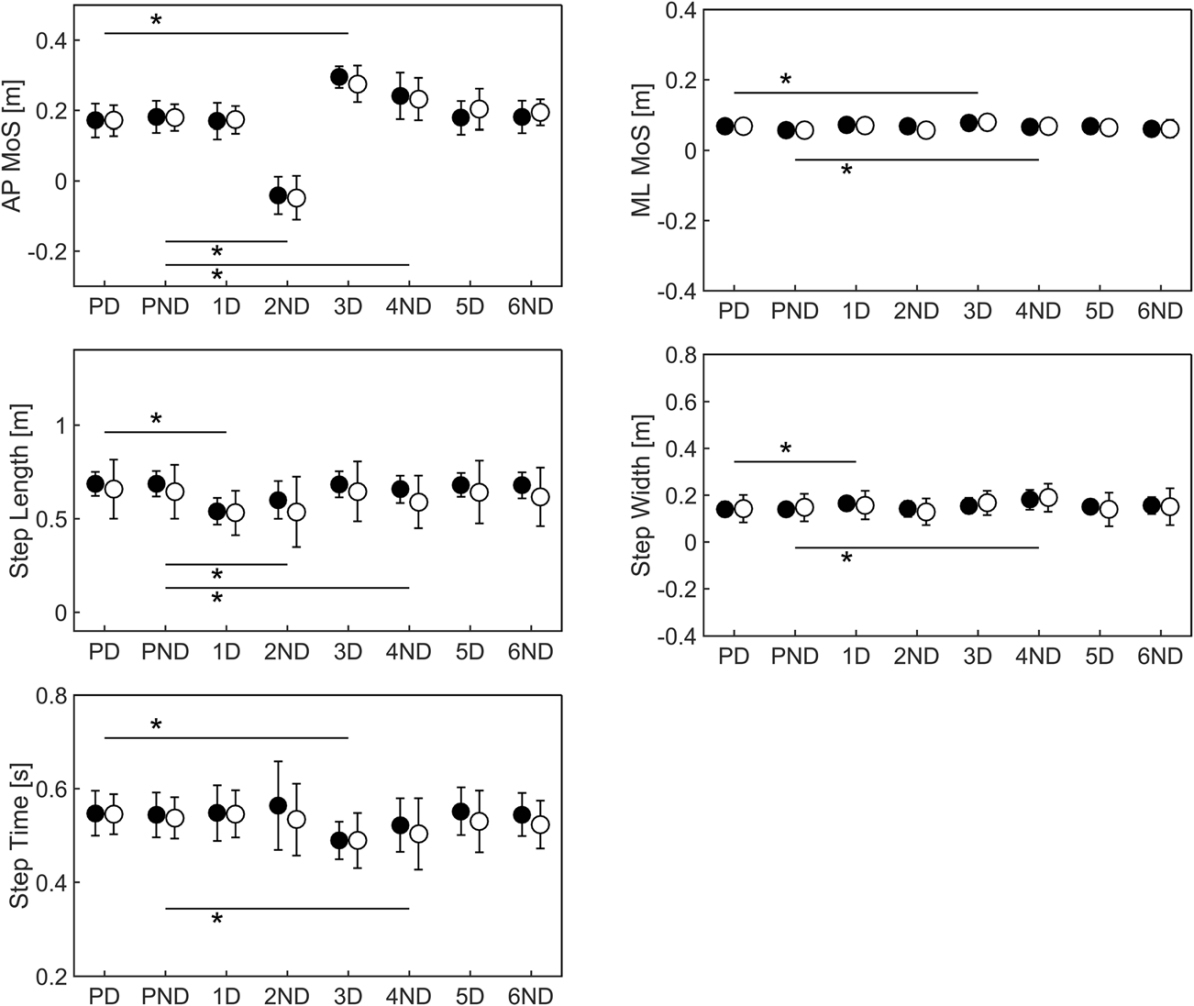
Mean and standard deviations of anterior-posterior (AP) and medio-lateral (ML) margins of stability (MoS), step length, width and time for steps for the deceleration (Dec) perturbations. Black dots represent for values young adults whereas white dots represent older adults. Significant differences between pre-perturbation (PD and PND) and post-perturbation (1D to 6ND) steps are indicated with *

**Fig. 8:**
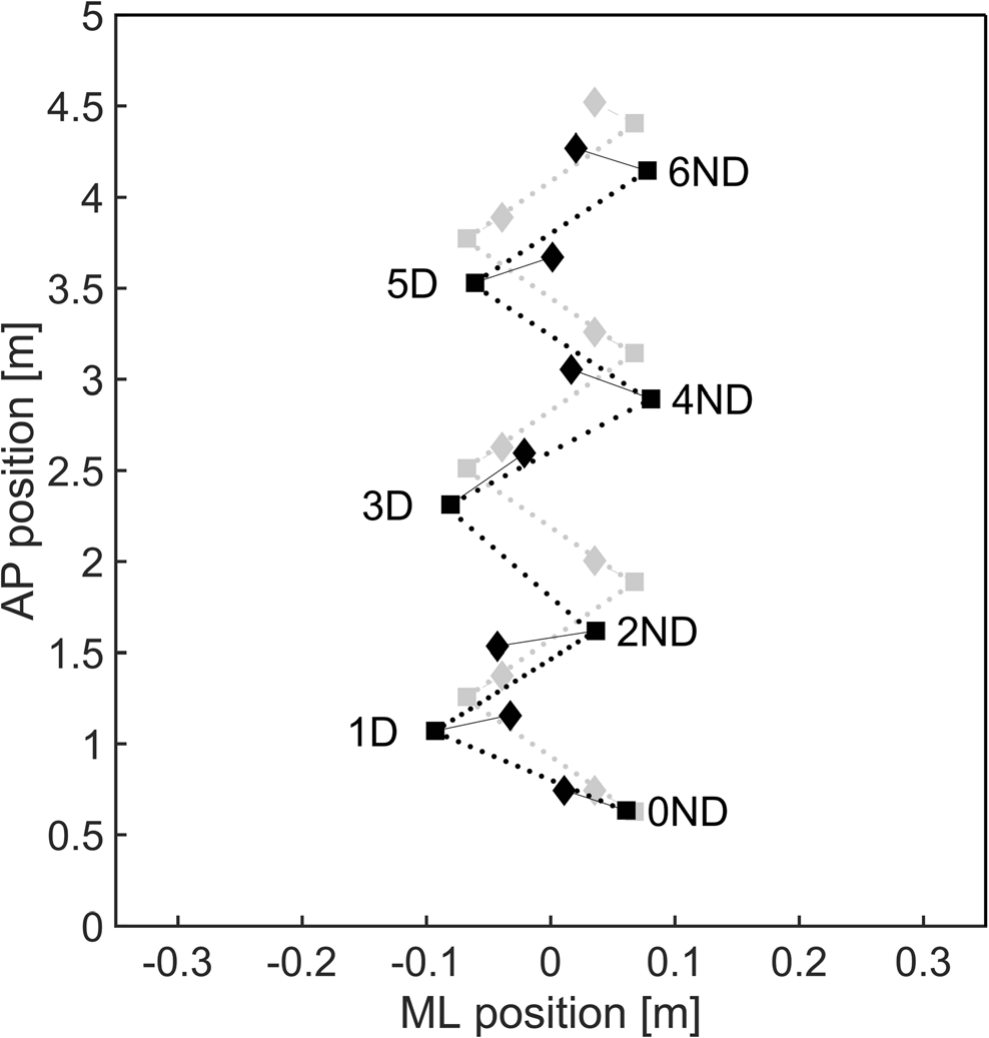
A schematic representation of a typical response (black) to a deceleration perturbation as compared to baseline walking (gray). Squares represent ML (step width) and AP (step length) foot placement whereas the line density is an indication of the time elapsed (step time) between consecutive steps. Margins of stability in the ML and AP direction are indicated by the diamonds. The perturbation was triggered at step 0ND

Mixed-model ANOVAs for the deceleration perturbation revealed significant main effects for all Steps on all gait parameters, while no significant Group or Group × Steps interaction effects were found. Post hoc analyses revealed a reduction to negative AP MoS (i.e. instability in the backward direction) at step 2ND (Fig. 7 and Supplementary Material Table S1). During step 3D to 5D, AP MoS was increased to values larger than those at baseline. A significant reduction in step time was found during step 3D and 4ND, whereas step lengths reduced during step 1D, 2ND, 4D and 6ND. Moreover, both ML MoS (step 3D and 4ND) and step width (1D, 3D and 4ND) increased. Figure 8 shows the relation between gait stability and spatio-temporal parameters for a typical deceleration perturbation response. Results on the analyses of the other perturbation types can be found in the Supplementary Material (Table S1 and Fig. S2).

## Discussion

We developed a gait perturbation protocol containing two platform, two belt, and two sensory perturbations. Our main aim was to evaluate which perturbation type affected stability the most in young and older adults. We found little differences in our groups of participants. However, the results showed that all mechanical perturbations effectively altered the gait pattern in both young and older adults while the sensory perturbations did not affect the gait pattern. The contralateral sway and deceleration perturbation appeared most challenging. Visual and auditory perturbations did not affect the gait pattern. This is in line with previous work, which showed that low light conditions did not affect spatio-temporal parameters [33, 34]. To our knowledge, auditory perturbations by means of acoustic startles have not been investigated previously.

The contralateral sway perturbation (i.e. platform movement to the right at left heel strike or the left at right heel strike) induced BoS movement towards the XCoM and thus ML MoS decreased. Consequently, the majority of the participants were required to take a cross-step to prevent falling. Following the initial perturbation response, ML MoS was recovered by taking faster, shorter and wider steps. Due to the adaptations in step length and step time, AP MoS increased as well [23]. Likewise, the deceleration perturbation reduced the distance between the border of the BoS and the XCoM in the backward direction and thus AP MoS initially decreased. Again, stability was recovered by taking faster, shorter, and wider steps. Previous work from Hof and colleagues (2010) reported comparable perturbation responses after ML waist-pushes. By definition, a BoS perturbation is expected to have a similar effect on ML MoS as a CoM perturbation in the opposite direction. Indeed, Hof and colleagues’ (2010) waist-pushes to the left at left heel strike were more challenging as compared to left pushes at right heel strike. The fact that acceleration perturbations appeared less challenging as compared to decelerations has been demonstrated previously in younger adults but to our knowledge not in older adults [19]. Ilmane and colleagues (2015) showed that the initial effect of the acceleration perturbations was larger compared to the deceleration, but recovery from the deceleration perturbations took much longer (up to four steps compared to one for the acceleration perturbation). The reduction in XCoM induced by the deceleration perturbation is extra challenging as one needs to maintain forward velocity to keep up with the treadmill speed.

While contralateral sway and deceleration perturbations evoked the largest responses, this does not necessarily mean that ipsilateral and acceleration perturbations should not be included in perturbation-based gait assessment. However, by applying more challenging perturbations, the (in)ability to adequately recover may be more profound and hence the perturbation response may be more sensitive to discriminate between fallers and non-fallers. The question whether the contralateral sway or deceleration perturbation is most challenging is more difficult to answer. Deviation from baseline (6S) was more than twice as large for the deceleration (0.52 ± 0.12 m) compared to the contralateral sway perturbation (0.22 ± 0.03 m). However, the fact that an ML change in *position* was induced by the sway perturbation as opposed to an AP change in *velocity* by the deceleration perturbation limits direct comparison. In a recent study, McIntosh et al. (2016) used ML and AP overground platform perturbations in young and older adults and found that contralateral sway perturbations were most challenging, followed by ipsilateral sway and then forward-backward perturbations. However, they quantified perturbation response by CoM displacement and velocity, while ignoring its relation to the BoS. Hence, it is unknown to what extent stability was affected. Additionally, whether ML or AP perturbations are more challenging may be patient-specific as a result of individual risk factors for falls such as decline in muscle strength or ineffective stepping strategies [1]. Therefore, including both contralateral sway and deceleration perturbations in gait stability assessment might give a more complete representation of one’s ability to resist or recover from a gait perturbation.

Successful recovery from a perturbation is determined by the combination of stability prior and in response to the perturbation. By proactively increasing gait stability, one might reduce the effect of the perturbation and minimize risk of falling [44–46]. Although we did not aim to evaluate such adaptations, the data revealed that participants proactively adapted their gait pattern. Gait adaptations were perturbation type specific but did not differ between age groups. Of interest would be to investigate whether more frail elderly show similar proactive gait adaptations and whether these adaptions are indicative of fall risk.

In contrast to our expectations based on previous studies [6, 7, 44, 47], we did not find any differences in perturbation effects and recovery responses between young and older adults. This may be explained by the fact that most of our older adults were recruited through fitness classes and therefore very fit and healthy. This potential selection bias was confirmed by the non-significant differences in steady state local dynamic stability during baseline walking, which is known to decrease with age [41–43]. Furthermore, differences between young and older adults in upper body movement were not included in our analyses. Previous work shows, for example, that arm movement strategies following trip perturbations are affected by ageing [48]. However, such differences should have been reflected in our gait stability measures, including full body CoM movement, which did not differ. Moreover, the perturbation intensities may have been too low to provoke responses close to the individuals’ boundaries. For example, McIntosh and colleagues (2016) used 15 cm ML platform excursions to discriminate between young and older adults as opposed to 5 cm in this study. Decelerations of 8 m/s^2^ (as opposed to our 2.43–5.13 m/s^2^) were used to distinguish fallers from non-fallers [49]. The perturbation intensities in this study were chosen such that a fall would not be induced, which we believe is preferable in clinical practice, but higher intensities might be required to reveal subtle group differences. Additionally, within this fit group more sensitive outcome measures may have been required to discriminate between young and older adults. For example, evaluation of trunk kinematics may have been of added value [50].

## Conclusion

No differences between young and older adults were found in the recovery response to medio-lateral platform, anterior-posterior belt, and sensory perturbations. However, our results revealed that contralateral sway and deceleration perturbations show most potential in disturbing the gait pattern in young and healthy older adults. Therefore, including these specific perturbation types in perturbation-based gait assessment may be preferred over ipsilateral sway, acceleration, visual or auditory perturbations. Further investigation including comparison between older adults with and without history of falls and possibly at higher intensities is required to see if and how perturbation-based gait assessment can be used to identify fall risk in the elderly population.

## Electronic supplementary material

ESM 1 (DOCX 47185 kb)

ESM 2 (SAV 72 kb)
