# Supplementary material for: Gait stability in response to platform, belt, and sensory perturbations in young and older adults
Source: Med Biol Eng Comput. 2018 Jun 27;56(12):2325–35. doi: 10.1007/s11517-018-1855-7 (PMC6245003; doi:10.1007/s11517-018-1855-7)

**Supplementary Material**

**Table S1** Paired-samples *t*-tests. Dominant post-perturbation steps (1D, 3D and 5D) were compared with the average dominant pre-perturbation step whereas non-dominant post-perturbation steps (2ND, 4ND, 6ND) were compared with the average non-dominant pre-perturbation step. Significantly different effects are printed in bold.

| Perturbation  type | Step | ML MoS | |  | AP MoS | |  | Step time | |  | Step length | |  | Step width | |
| --- | --- | --- | --- | --- | --- | --- | --- | --- | --- | --- | --- | --- | --- | --- | --- |
|  |  | *t* | *p* |  | *t* | *p* |  | *t* | *p* |  | *t* | *p* |  | *t* | *p* |
| SwyI | 1D | -2.753 | 0.082 |  | 0.577 | 1.000 |  | **7.429** | **<0.001** |  | **9.667** | **<0.001** |  | **-13.321** | **<0.001** |
|  | 2ND | **-7.873** | **<0.001** |  | **-9.942** | **<0.001** |  | **6.287** | **<0.001** |  | **4.815** | **0.001** |  | **-7.928** | **<0.001** |
|  | 3D | 1.120 | 1.000 |  | **-5.464** | **<0.001** |  | 2.521 | 0.132 |  | -0.342 | 1.000 |  | -0.378 | 1.000 |
|  | 4ND | -2.237 | 0.234 |  | -2.950 | 0.054 |  | 2.379 | 0.176 |  | 0.745 | 1.000 |  | -2.610 | 0.110 |
|  | 5D | -1.669 | 0.680 |  | **-4.315** | **0.003** |  | 2.473 | 0.146 |  | 0.684 | 1.000 |  | **-3.289** | **0.026** |
|  | 6ND | 1.169 | 1.000 |  | -1.815 | 0.523 |  | 0.868 | 1.000 |  | 1.511 | 0.895 |  | 0.163 | 1.000 |
| SwyC | 1D | **26.264** | **<0.001** |  | 0.533 | 1.000 |  | -0.862 | 1.000 |  | **3.309** | **0.025** |  | **14.084** | **<0.001** |
|  | 2ND | **4.202** | **0.004** |  | **-4.698** | **0.001** |  | **4.912** | **0.001** |  | **4.133** | **0.004** |  | 2.636 | 0.104 |
|  | 3D | **-10.245** | **<0.001** |  | **-5.351** | **<0.001** |  | **3.357** | **0.022** |  | **4.411** | **0.002** |  | **-11.539** | **<0.001** |
|  | 4ND | **-3.624** | **0.013** |  | **-6.005** | **<0.001** |  | **4.419** | **0.002** |  | 2.528 | 0.130 |  | **-6.240** | **<0.001** |
|  | 5D | **-3.631** | **0.012** |  | -2.731 | 0.085 |  | **3.666** | **0.011** |  | **3.152** | **0.035** |  | **-3.687** | **0.011** |
|  | 6ND | -2.158 | 0.273 |  | -2.919 | 0.057 |  | 1.584 | 0.790 |  | **3.591** | **0.014** |  | **-3.121** | **0.037** |
| Acc | 1D | -0.312 | 1.000 |  | -0.846 | 1.000 |  | **4.715** | **0.002** |  | **-11.268** | **<0.001** |  | 0.884 | 1.000 |
|  | 2ND | **-7.006** | **<0.001** |  | **-21.256** | **<0.001** |  | **9.069** | **<0.001** |  | **4.303** | **0.004** |  | **-6.164** | **<0.001** |
|  | 3D | **-5.249** | **0.001** |  | 1.581 | 0.808 |  | -0.175 | 1.000 |  | **3.462** | **0.021** |  | **-7.935** | **<0.001** |
|  | 4ND | 0.070 | 1.000 |  | -0.386 | 1.000 |  | -0.885 | 1.000 |  | **-6.823** | **<0.001** |  | 0.434 | 1.000 |
|  | 5D | -1.235 | 1.000 |  | **-5.774** | **<0.001** |  | **3.717** | **0.012** |  | **-3.516** | **0.019** |  | -1.954 | 0.417 |
|  | 6ND | 1.021 | 1.000 |  | -1.579 | 0.811 |  | -0.413 | 1.000 |  | -0.269 | 1.000 |  | 0.098 | 1.000 |
| Dec | 1D | -1.555 | 0.844 |  | 0.053 | 1.000 |  | -0.120 | 1.000 |  | **12.099** | **<0.001** |  | **-3.570** | **0.017** |
|  | 2ND | -1.728 | 0.627 |  | **19.769** | **<0.001** |  | -0.804 | 1.000 |  | **7.342** | **<0.001** |  | 1.058 | 1.000 |
|  | 3D | **-4.827** | **0.001** |  | **-13.325** | **<0.001** |  | **8.554** | **<0.001** |  | 0.606 | 1.000 |  | -2.638 | 0.112 |
|  | 4ND | **-4.602** | **0.002** |  | **-5.446** | **<0.001** |  | **3.548** | **0.018** |  | **4.712** | **0.002** |  | **-5.573** | **<0.001** |
|  | 5D | 1.177 | 1.000 |  | -2.503 | 0.146 |  | 0.810 | 1.000 |  | 1.686 | 0.675 |  | -1.363 | 1.000 |
|  | 6ND | -1.760 | 0.593 |  | -1.294 | 1.000 |  | 1.519 | 0.897 |  | 2.204 | 0.261 |  | -1.582 | 0.807 |
| Viz | 1D | -1.509 | 0.898 |  | -1.785 | 0.553 |  | - | - |  | - | - |  | - | - |
|  | 2ND | -0.536 | 1.000 |  | -1.694 | 0.651 |  | - | - |  | - | - |  | - | - |
|  | 3D | -0.446 | 1.000 |  | 0.817 | 1.000 |  | - | - |  | - | - |  | - | - |
|  | 4ND | -0.922 | 1.000 |  | 1.602 | 0.765 |  | - | - |  | - | - |  | - | - |
|  | 5D | -1.281 | 1.000 |  | -0.389 | 1.000 |  | - | - |  | - | - |  | - | - |
|  | 6ND | -0.474 | 1.000 |  | 0.942 | 1.000 |  | - | - |  | - | - |  | - | - |
| Aud | 1D | 1.454 | 0.985 |  | - | - |  | - | - |  | - | - |  | - | - |
|  | 2ND | 1.212 | 1.000 |  | - | - |  | - | - |  | - | - |  | - | - |
|  | 3D | -0.170 | 1.000 |  | - | - |  | - | - |  | - | - |  | - | - |
|  | 4ND | -0.210 | 1.000 |  | - | - |  | - | - |  | - | - |  | - | - |
|  | 5D | **3.218** | **0.030** |  | - | - |  | - | - |  | - | - |  | - | - |
|  | 6ND | 0.243 | 1.000 |  | - | - |  | - | - |  | - | - |  | - | - |

**Figure S1** Mean and standard deviations of anterior-posterior (AP) and medio-lateral (ML) margins of stability (MoS), step length, width and time for steps for the ipsi-lateral sway (SwyI), acceleration (Acc), visual (Viz) and auditory (Aud) perturbations. Black dots represent for young adults whereas white dots represent older adults. Significant differences between pre- and post-perturbations steps are indicated with *.

**SwyI**


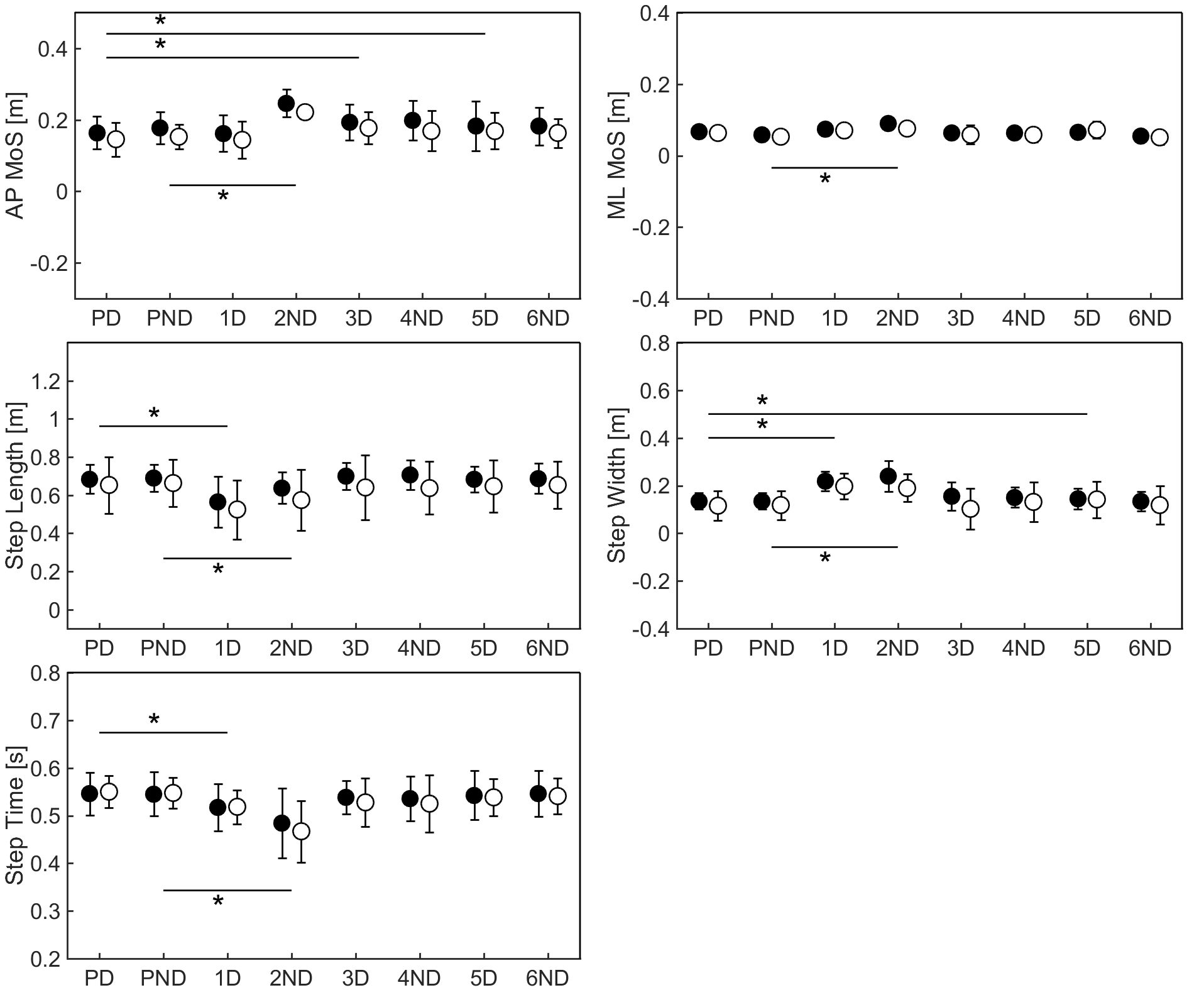


**Acc**
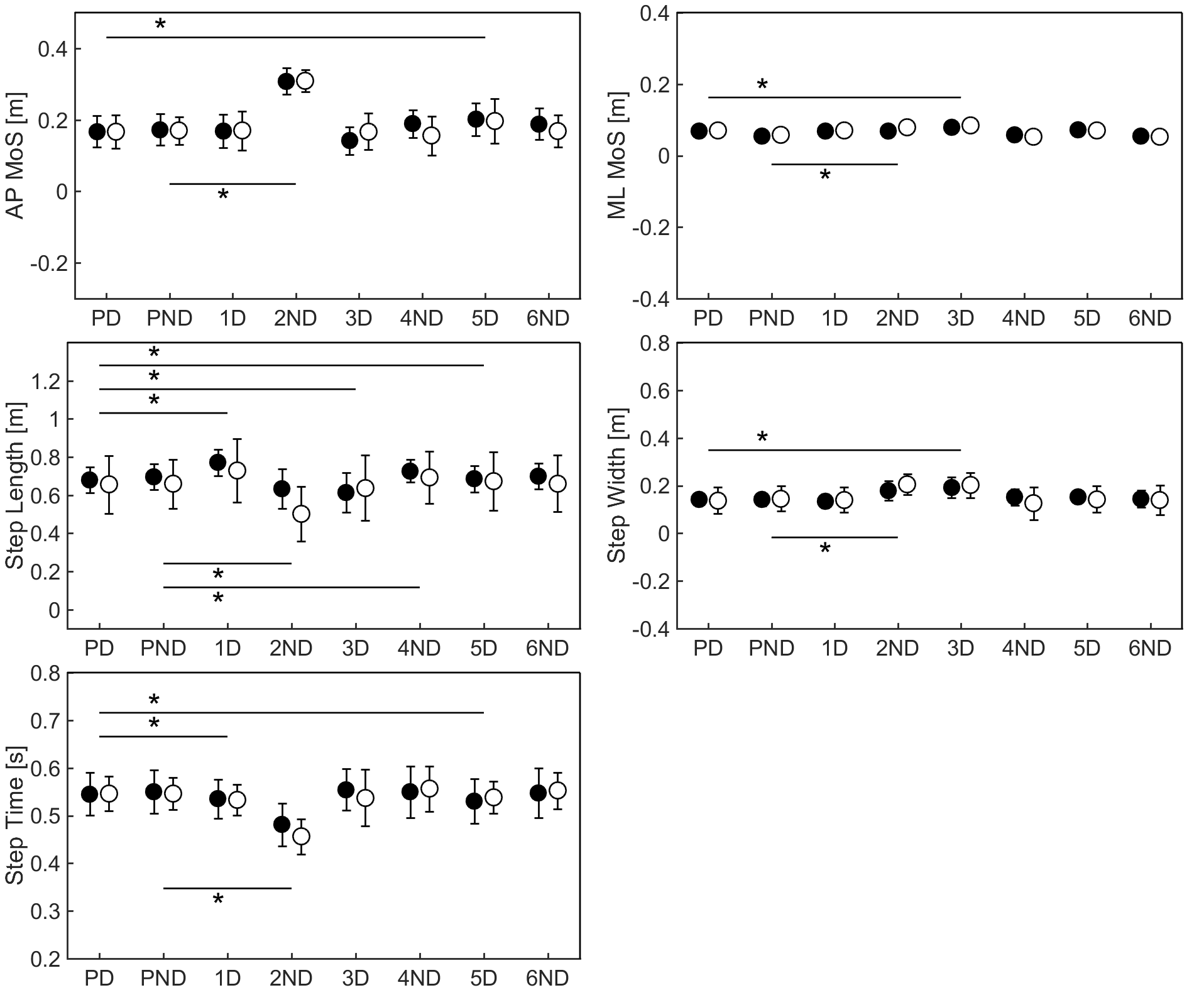


**Viz**


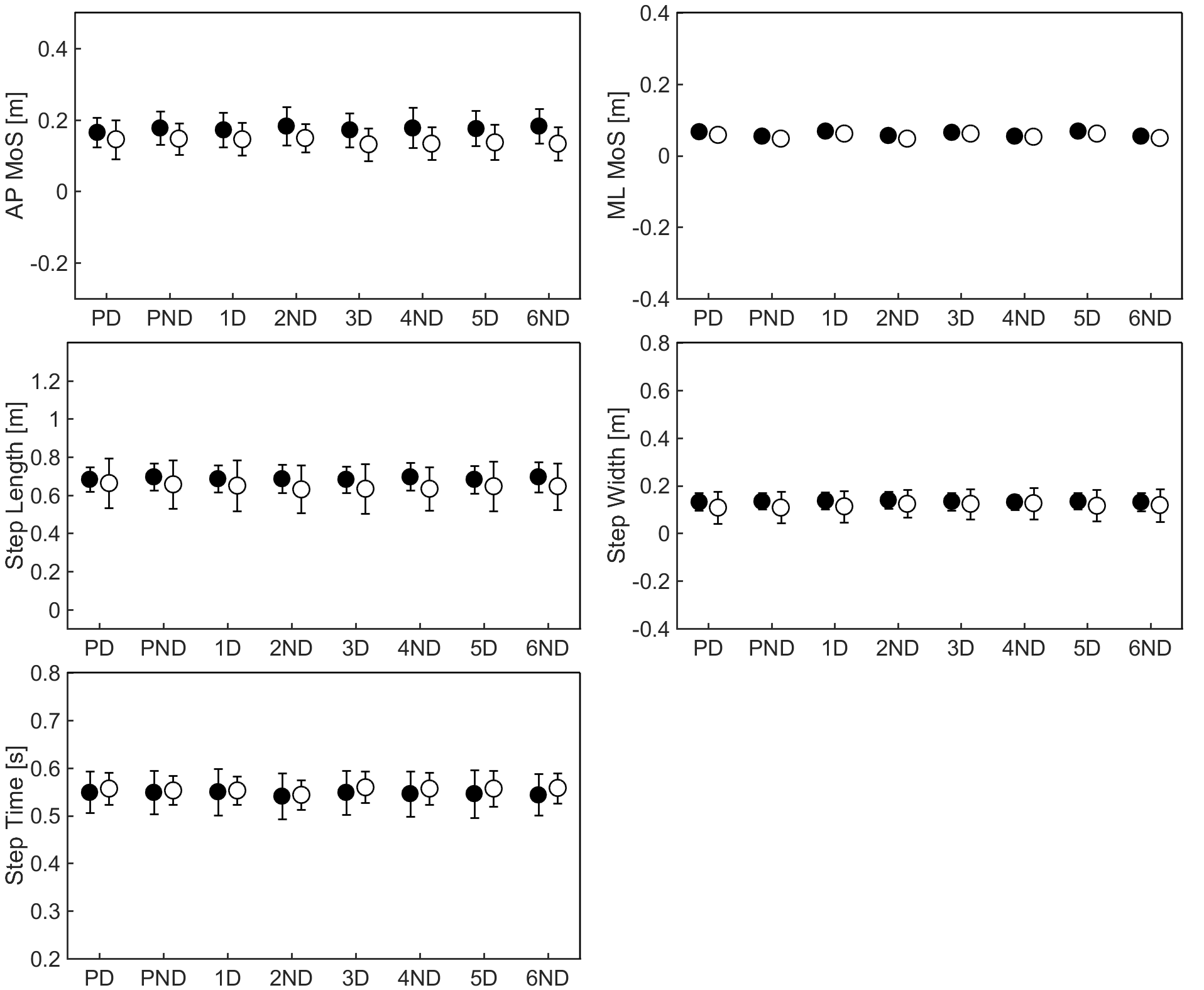


**Aud**


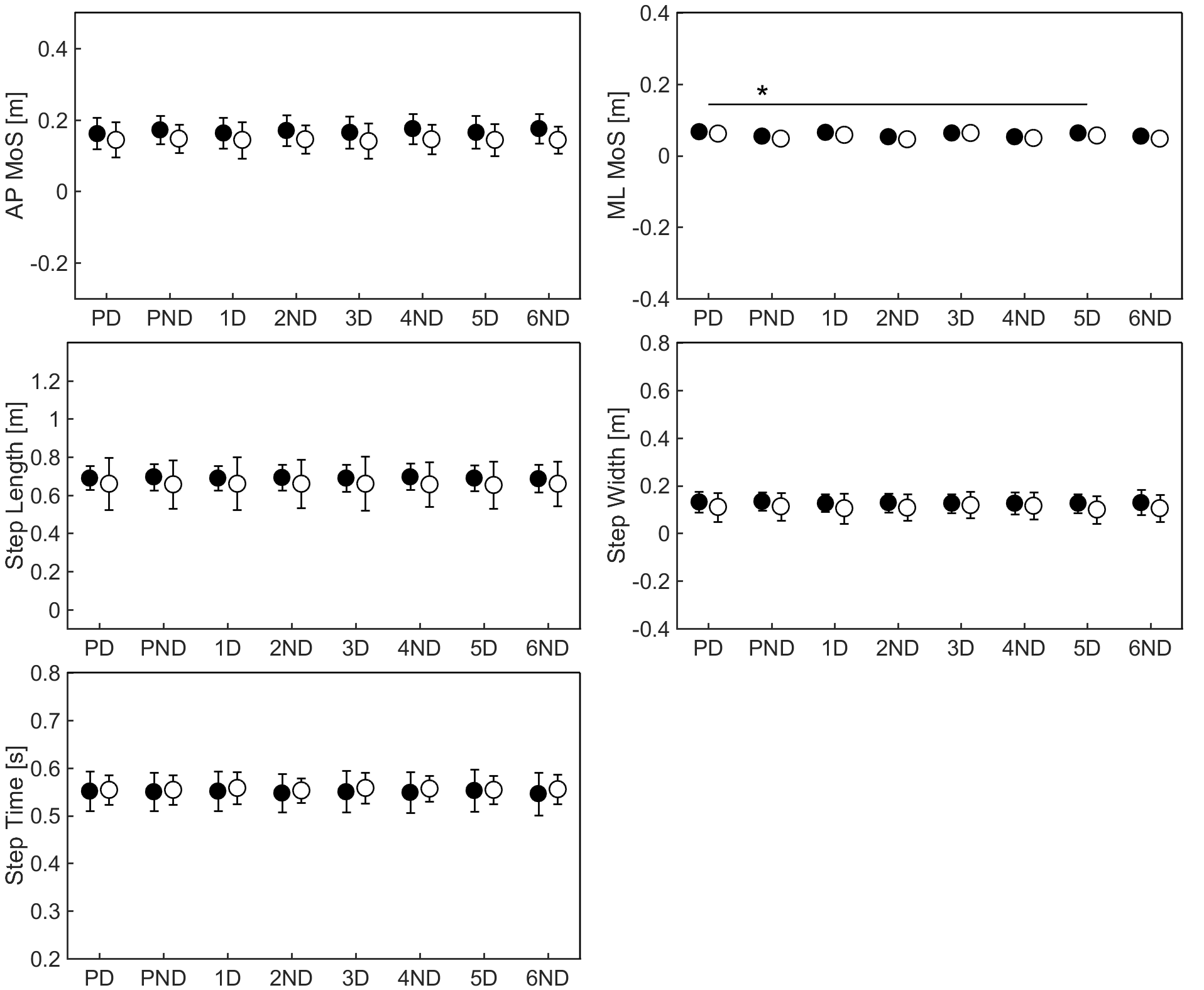

Supplement: Supplementary file 1 — (DOCX 47185 kb) [file 11517_2018_1855_MOESM1_ESM.docx]
